# Global transcriptome-wide analysis of CIK cells identify distinct roles of IL-2 and IL-15 in acquisition of cytotoxic capacity against tumor

**DOI:** 10.1186/1755-8794-7-49

**Published:** 2014-08-09

**Authors:** Wenju Wang, Mingyao Meng, Yayong Zhang, Chuanyu Wei, Yanhua Xie, Lihong Jiang, Chunhui Wang, Fang Yang, Weiwei Tang, Xingfang Jin, Dai Chen, Jie Zong, Zongliu Hou, Ruhong Li

**Affiliations:** 1 Yan’an Affiliated Hospital of Kunming Medical University, Kunming 650051, Yunnan, People’s Republic of China; 2 Kunming Medical University, Kunming 650050, Yunnan, People’s Republic of China; 3 Novel Bioinformatics Co., Ltd, Shanghai, China

**Keywords:** CIK cells, Interleukin 2, Interleukin 15, Deep sequencing, Transcriptome

## Abstract

**Background:**

Cytokine-induced killer (CIK) cells are an emerging approach of cancer treatment. Our previous study have shown that CIK cells stimulated with combination of IL-2 and IL-15 displayed improved proliferation capacity and tumor cytotoxicity. However, the mechanisms of CIK cell proliferation and acquisition of cytolytic function against tumor induced by IL-2 and IL-15 have not been well elucidated yet.

**Methods:**

CIK_IL-2_ and CIK_IL-15_ were generated from peripheral blood mononuclear cells primed with IFN-γ, and stimulated with IL-2 and IL-15 in combination with OKT3 respectively. RNA-seq was performed to identify differentially expressed genes, and gene ontology and pathways based analysis were used to identify the distinct roles of IL-2 and IL-15 in CIK preparation.

**Results:**

The results indicated that CIK_IL-15_ showed improved cell proliferation capacity compared to CIK_IL-2_. However, CIK_IL-2_ has exhibited greater tumor cytotoxic effect than CIK_IL-15_. Employing deep sequencing, we sequenced mRNA transcripts in CIK_IL-2_ and CIK_IL-15_. A total of 374 differentially expressed genes (DEGs) were identified including 175 up-regulated genes in CIK_IL-15_ and 199 up-regulated genes in CIK_IL-2_. Among DEGs in CIK_IL-15_, Wnt signaling and cell adhesion were significant GO terms and pathways which related with their functions. In CIK_IL-2_, type I interferon signaling and cytokine-cytokine receptor interaction were significant GO terms and pathways. We found that the up-regulation of Wnt 4 and PDGFD may contribute to enhanced cell proliferation capacity of CIK_IL-15_, while inhibitory signal from interaction between CTLA4 and CD80 may be responsible for the weak proliferation capacity of CIK_IL-2_. Moreover, up-regulated expressions of CD40LG and IRF7 may make for improved tumor cytolytic function of CIK_IL-2_ through type I interferon signaling.

**Conclusions:**

Through our findings, we have preliminarily elucidated the cells proliferation and acquisition of tumor cytotoxicity mechanism of CIK_IL-15_ and CIK_IL-2_. Better understanding of these mechanisms will help to generate novel CIK cells with greater proliferation potential and improved tumor cytolytic function.

## Background

Cancer is still a leading cause of diseases related death all over the world. It was estimated that 7.6 million people were dead from various types of cancer in 2008, and the figure will continue to rise to 13.1 million in 2030 [1]. Fortunately, significant progress has been made to develop better approaches to prevent, diagnose and treat cancer in the past several years. These advances have made more people survive with their cancer today. However, these new approaches are not completely effective to all of cancers, and side effects were brought by some of treatments. Among these advances, immunotherapy has shown its large potential in cancer therapy. Cytokine-induced killer (CIK) cells, a subset of T lymphocytes with a natural killer T cell phenotype, have been proven to be effective to most of tumors in vitro and in vivo [2]. CIK cells exhibit potent cytolytic activities against tumor cells with minimal adverse effects. CIK cells are prepared from peripheral blood mononuclear cells (PBMCs) by priming with IFN-γ, and maintained with monoclonal antibody against CD3 (OKT3) and interleukin-2 in the following days [3]. During the generation of CIK cells, monoclonal antibody against CD3 provided mitogenic signals to T lymphocytes. Priming with IFN-γ is to activate the monocytes which provide contact-dependent (CD58/LFA-3) and soluble (IL-12) crucial signals promoting generation of autophagy and antigen cross-presentation [4]. In following bulk culture, IL-2 promotes T cell proliferation, survival and acquisition of cytolytic effector function.

IL-15 is a cytokine which stimulate growth of NK, NKT cells and activated T lymphocytes in peripheral, and it has similar biological properties with IL-2 in innate immunity [5]. Studies have suggested that IL-15 bind to subunits of IL-2 receptor and common gamma chain [6]. Because IL-15 and IL-2 share common signaling components, they mediate a series of similar signaling events. These events include activation of the Janus kinase (Jak) and STAT pathways. The two cytokines both can facilitate the induction of tumor toxic effector T cells and proliferation of NK cells. However, IL-15 and IL-2 are differed in their cDNA/protein sequence and contribute differently to T cell-mediated immune response [6]. Although IL-2 is a growth and survival factor, it plays important role in Fas-mediated activation-induced cell death (AICD) of CD4 T cell. In contrast, IL-15 promotes the survival of T lymphocytes by inhibiting IL-2-mediated CD4^+^ T cell AICD [7].

In our previous study, we have shown that CIK cells stimulated with combination of IL-2 and IL-15 exhibited enhanced cytotoxic capacity against lung cancer both in vitro and in vivo. Interestingly, we found that CIK cells activated with IL-2 and IL-15 could up-regulate the expression levels of IFN-γ and TNF-α in vivo compared to CIK cell stimulated with IL-2 alone [8]. In order to identify the roles of IL-2 and IL-15 during induction of tumor toxic function of CIK cells, we performed comparative transcriptome analysis between CIK cells prepared with IL-15 and IL-2 respectively by Ion PI mRNA sequencing (RNA-seq) for the first time. The mRNAs isolated from CIK_IL-15_ cells and CIK_IL-2_ cells were transcribed into cDNAs which were applied to deep sequencing. The results of RNA-seq were analyzed by a series of bioinformatic methods including mapping, gene differential expression analysis, gene ontology (GO) and pathway analysis. Our finding will provide evidence for optimizing the CIK cell propagation strategy which produces more effective CIK cells against tumor.

## Methods

### Cell lines and reagents

Human lung adenocarcinoma (SPC-A-1 cells) and gastric tumor cells (BGC823) were obtained from Chinese Type Culture Collection (Shanghai, PR China). FITC conjugated anti-CD56 antibody and R-phycoerythrin conjugated anti-CD3 antibody used in identifying CIK phenotypic markers were purchased from BD Biosciences. The cell viability assay kit (Cell Counting Kit-8) was purchased from Dojindo, Molecular Technologies. Reagents for CIK cells generation including OKT3, IFN-γ, IL-2 and IL-15 were from Miltenyi Biotec. Experiments involving human peripheral blood were reviewed and approved by Bioethics Committee of Yan’an Affiliated Hospital of Kunming Medical University. Written informed consents have been given from all volunteers participated in this study.

### Generation of CIK_IL-2_ and CIK_IL-15_ (Standard protocols)

The Bioethics Committee of Yan’an Affiliated Hospital of Kunming Medical University has approved the investigation protocols to draw blood from healthy volunteers after written informed consent for the purposes of preparation CIK cells against tumor and deep sequencing. CIK cells were prepared from PBMCs which were isolated by standard Ficoll separation. PBMCs were cultured in RPMI 1640 growth medium at a density of 5 × 10^6^ cells/mL. The RPMI 1640 growth medium for CIK contained 10% FBS, 2% L-glutamine and antibiotics. The generation of CIK cells was primed by adding 1000 U/mL IFN-γ on day 0 and 100 ng/mL anti-CD3 antibody and 500 U/mL IL-2 or 10 ng/mL IL-15 within the following 15 days of culture. The CIK cells were propagated every 5 days with RPMI 1640 growth medium supplemented with anti-CD3 antibody and IL-2 or IL-15 respectively [9]. The CIK cells were expanded for 15 days and analyzed every 5 days.

### Cytotoxicity assay based on CCK-8

After co-culture with CIK cells for 48 hours, the cell viabilities of two tumor cells were determined by CCK-8 based methods. Briefly, 10uL of CCK-8 solution was added in each well, and the plates were incubated at 37°C for 2–4 hours. After incubation, the absorbance of each well was read by a spectrophotometer at 450 nm. Each sample for one treatment was calculated by values from 5 independent samples.

### RNA extraction and quality control

Total RNA was extracted from each sample using TRIzol Reagent (Life technologies, USA) according to the protocol from manufacturer. The concentration of each sample was measured by NanoDrop 2000 (Thermo Scientific, USA). The quality was assessed by the Agilent2200 (Agilent, USA).

### Whole transcriptome libraries preparation and deep sequencing

The sequencing library of each RNA sample was prepared by using Ion Total RNA-Seq Kit v2 according to the protocol provided by manufacturer (Life technologies, USA). Briefly, poly(A)-containing mRNA was purified from 5 ug total RNA with Dynabeads (Life technologies, USA). The mRNA was fragmented using RNaseIII and purified. The fragmented RNA was hybrized and ligated with Ion adaptor. The RNA fragments were reverse-transcribed and amplified to double-stranded cDNA. Then, the amplified cDNA was purified by magnetic bead based method, and the molar concentration was determined for each cDNA library. Emulsion PCR was performed using template of cDNA library. The Template-Positive Ion PI^TM^ Ion Sphere^TM^ Particles were enriched and loaded on the Ion PI^TM^ chip for sequencing.

### Filtering raw reads and mapping

The raw reads ≥50 bp which passed filtering were used for mapping. We used the Masplicing as our RNA-seq data mapping analysis tool whose core program is Bowtie that can identify the exon-exon splicing immediately and accurately [10].

### Identification of differentially expressed genes

We applied the DEseq to filter the differentially expressed genes for the CIK_IL-15_ and CIK_IL-2_ groups. After the statistical analysis, we selected the differentially expressed genes according to the FDR threshold (FDR < 0.05) [11].

### GO analysis

GO analysis was applied to analyze the main function of the differential expression genes according to the Gene Ontology which is the key functional classification of NCBI [12,13]. Generally, Fisher’s exact test and *χ*^2^ test were used to classify the GO category, and the false discovery rate (FDR) was calculated to correct the P-value, the smaller the FDR, the small the error in judging the p-value [14,15]. The FDR was defined as $\mathrm{FDR}=1-\frac{N_{k}}{T}$, where *N*_*k*_ refers to the number of Fisher’s test *P*-values less than *χ*^2^ test *P*-values. We computed *P*-values for the GOs of all the differential genes. The significant GO terms were defined as P value <0.05 and FDR <0.05. Concerning on the treatment of GO term redundancy, we have adopted strategy of filtering out terms by picking only one from each leaf-to-root path.

### Pathway analysis

Similarly, pathway analysis was used to find out the significant pathway of the differential genes according to KEGG, Biocarta and Reactome [10,16,17]. Still, we turn to the Fisher’s exact test and *χ*^2^ test to select the significant pathway, and the threshold of significance was defined by P-value and FDR. The significant pathway was identified by P value <0.05 and FDR < 0.05. The enrichment was calculated like the equation above [18-20].

### Gene-act-network

Use the KEGG database to build the network of genes according to the relationship among the genes, proteins and compounds in the database [21-25].

### Path-act-network

KEGG database has included metabolism, membrane transport, signal transduction, cell cycle pathways and information about interactions among them. The genes we have selected may involved in two or more signaling pathways. Because of the same genes in different pathways, overlappings between pathways were obvious. We picked the genes in enriched biological pathway and used Cytoscape for graphical representations of pathways [26].

### Co-expression network analysis

For each pair of genes, we calculate the Pearson Correlation and choose the significant correlation pairs (FDR < 0.01) to construct the network [27]. Within the network analysis, degree centrality is the most simplest and important measures of the centrality of a gene within a network that determine the relative importance. Degree centrality is defined as the link numbers one node has to the other [28]. Moreover, to study some properties of the networks, k-cores in graph theory were introduced as a method of simplifying graph topology analysis [29].

### Quantitative reverse-transcription PCR

All the qRT-PCR involved in this study was performed on the CFX96 Touch™ (BIORAD, USA). The first strand of cDNA was synthesized with adjusted concentration of RNA, and corresponding genes were amplified by employing EVA Green Supermix. All the primers used for qRT-PCR were obtained from GeneCopoeia (USA).

## Results

### Enhanced cell proliferation capacity of CIK_IL-15_ and superior tumor toxic effect of CIK _IL-2_

**Figure 1:**
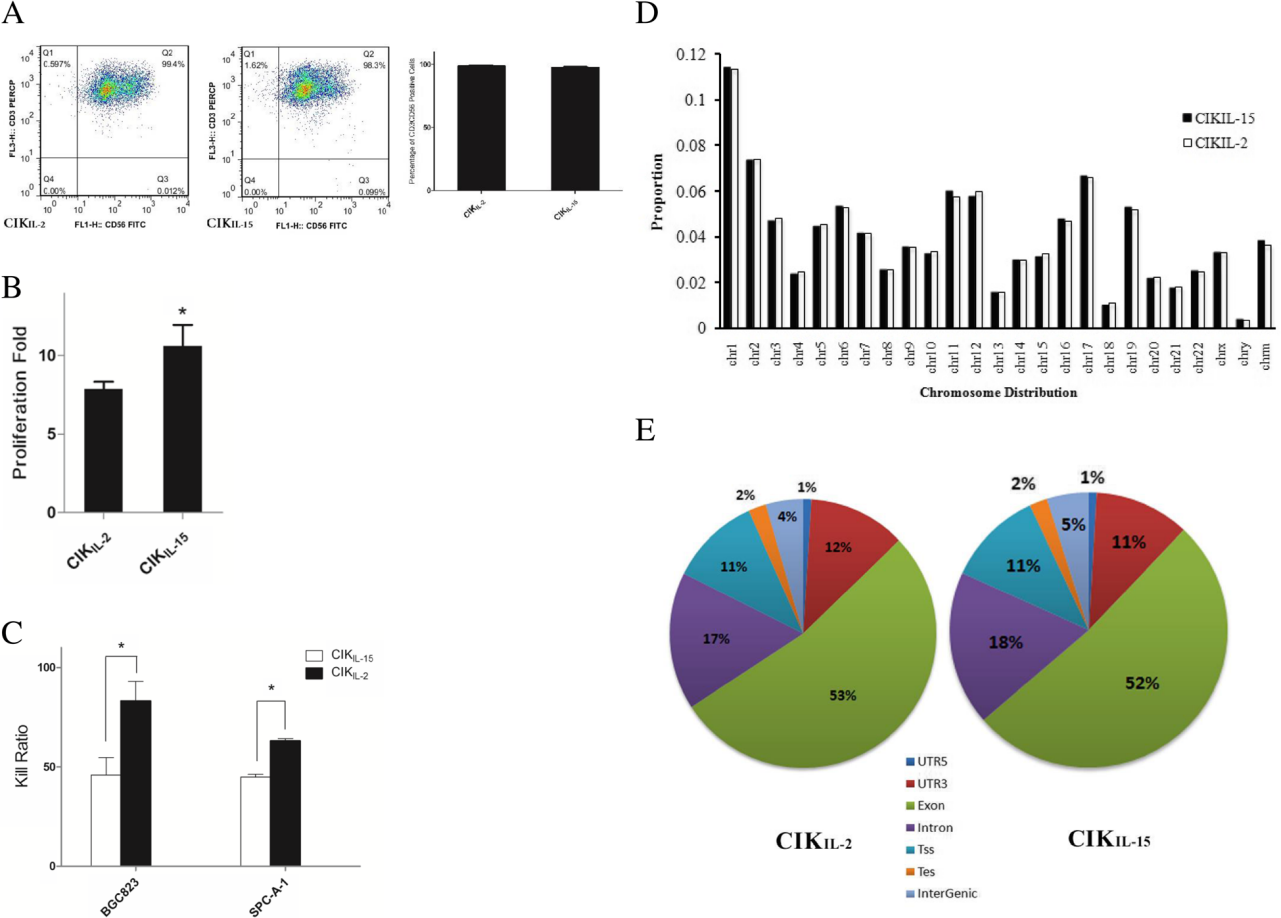
**A overview of phenotypes, functions and RNA-seq quality control of CIK**_**IL-15 **_**and CIK**_**IL-2**_**. (A)** Flow cytometric and statistical analysis of the proportion of CD3^+^CD56^+^ CIK cells. Numbers indicate the percentage of each subset. **(B)** Cell proliferation capacity assay of CIK_IL-15_ and CIK_IL-2_ based on automatic cell counting; **(C)** Detection of tumor cytotoxic effect of CIK_IL-15_ and CIK_IL-2_ against SPC-A-1 and BGC823; **(D)** Distribution of reads on chromosomes; **(E)** Percentage of mapped reads onto the regions of exons, introns, 5’-UTR, 3’-UTR, transcription start site (TSS), transcription end site (TES) and intergenic region.

CIK cells were generated from peripheral blood mononuclear cells of three healthy volunteers. The CIK_IL-15_ and CIK_IL-2_ cells were confirmed by flow cytometry with the phenotypes of CD3^+^CD56^+^. The results have demonstrated that the percentages of CD3^+^CD56^+^ cells were 98.80 ± 0.503% and 97.60 ± 0.603% respectively in CIK_IL-2_ and CIK_IL-15_ (Figure 1A). We determined the proliferation capacities of CIK_IL-15_ and CIK_IL-2_ by automatic cell counting. The result showed that CIK_IL-15_ displayed significantly higher proliferation capacity than CIK_IL-2_ (Figure 1B). To evaluate the tumor toxic effects of CIK_IL-15_ and CIK_IL-2_, we have chosen two types of tumor cell lines including human gastric tumor (BGC823) and human lung adenocarcinoma (SPC-A-1) as the targets in anti-tumor assay. After co-culture with CIK_IL-15_ and CIK_IL-2_ for 48 hours, the cell viabilities were measured for each type of tumor based on CCK-8 method. The results indicated that CIK_IL-2_ cells have shown greater cytotoxic potential against tumor than CIK_IL-15_ (Figure 1C). In order to investigate the distinct roles of IL-2 and IL-15 in CIK cell generation, we performed transcriptome-wide analysis of CIK_IL-2_ (n = 3) and CIK_IL-15_ (n = 3) by deep sequencing.

### Overview of sequencing data of RNA-seq analysis

Total raw reads among the six samples ranged from 19 to 34 million. The average of the GC content is approximately 49% for each sample. By a stringent quality check, more than 95% of the reads we obtained have a quality score of ≧Q20. The sequencing quality was analyzed by using RSeQC [30]. The raw sequence data have yielded about 2.2 gigabases (GB) of data per sample. About 1.58 ± 0.48 × 10^7^ reads (73.5% of the total raw reads) were mapped to human genome sequence in the six independent samples (Table 1) and 1.49 ± 0.48 × 10^7^ reads (69.5% of the total raw reads) were uniquely aligned to human genome. The mapping of the reads was performed by using MapSplice. Mapped reads in six independent samples were distributed consistently on the chromosomes (Figure 1D). We found that chromosome 1 has been matched the most reads and the least reads were found in chromosome Y. In the uniquely mapped reads, more than 50% of the reads were aligned at the transcript exon, 17% at the intron regions, 13% at the UTR regions and the remaining at TES (transcription end site), TSS (transcription start site) and intergenic regions (Figure 1E). Subsequently, we analyzed the aligned reads for transcript assembly, abundance evaluation and normalization. After annotation, there were 3,6267 transcripts annotated with known function Additional file 1. In order to quantify the expression levels of the transcripts, the RNA-seq data was normalized to RPKM values.

**Table 1:** Statistics of raw and mapped reads from RNA-seq analysis of CIK cells stimulated by IL-15 and IL-2 respectively

	**CIK**_**IL-15**_**-1**	**CIK**_**IL-15**_**-2**	**CIK**_**IL-15**_**-3**	**CIK**_**IL-2**_**-1**	**CIK**_**IL-2**_**-2**	**CIK**_**IL-2**_**-3**
**Raw reads**	19810412	19235209	21693091	22108193	24539599	21801487
**Unmapped reads**	5198537	4707536	5509521	6426050	6846138	5934418
**Mapped reads (Rate)**	14611875 (0.74)	14527675 (0.76)	16183570 (0.75)	15682144 (0.71)	17693461 (0.72)	15867070 (0.73)
**Unique mapping (Rate)**	13874295 (0.70)	13786260 (0.72)	15299443 (0.71)	14820672 (0.67)	16748163 (0.68)	15024084 (0.69)
**Repeat mapping**	737580	741413	884127	861471	945298	842985

### Differential gene expression profiles of CIK_IL-15_ and CIK_IL-2_ and GO analysis

**Figure 2:**
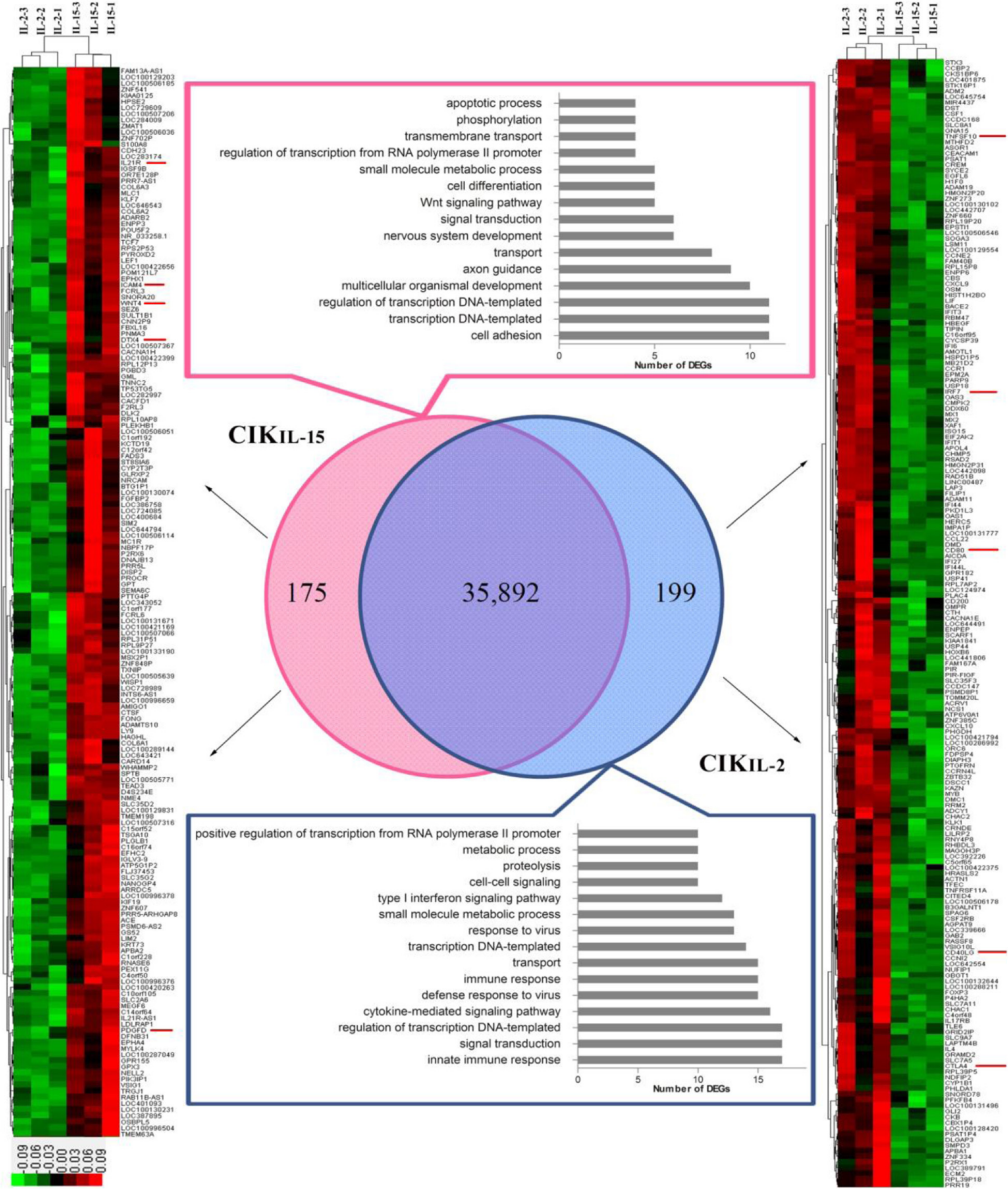
**Clustering of differentially expressed genes in CIK**_**IL-15 **_**and CIK**_**IL2 **_**and multiple DEGs involved GO terms.** The genes included for further analysis were labeled with red line by the sides of their gene symbols.

**Figure 3:**
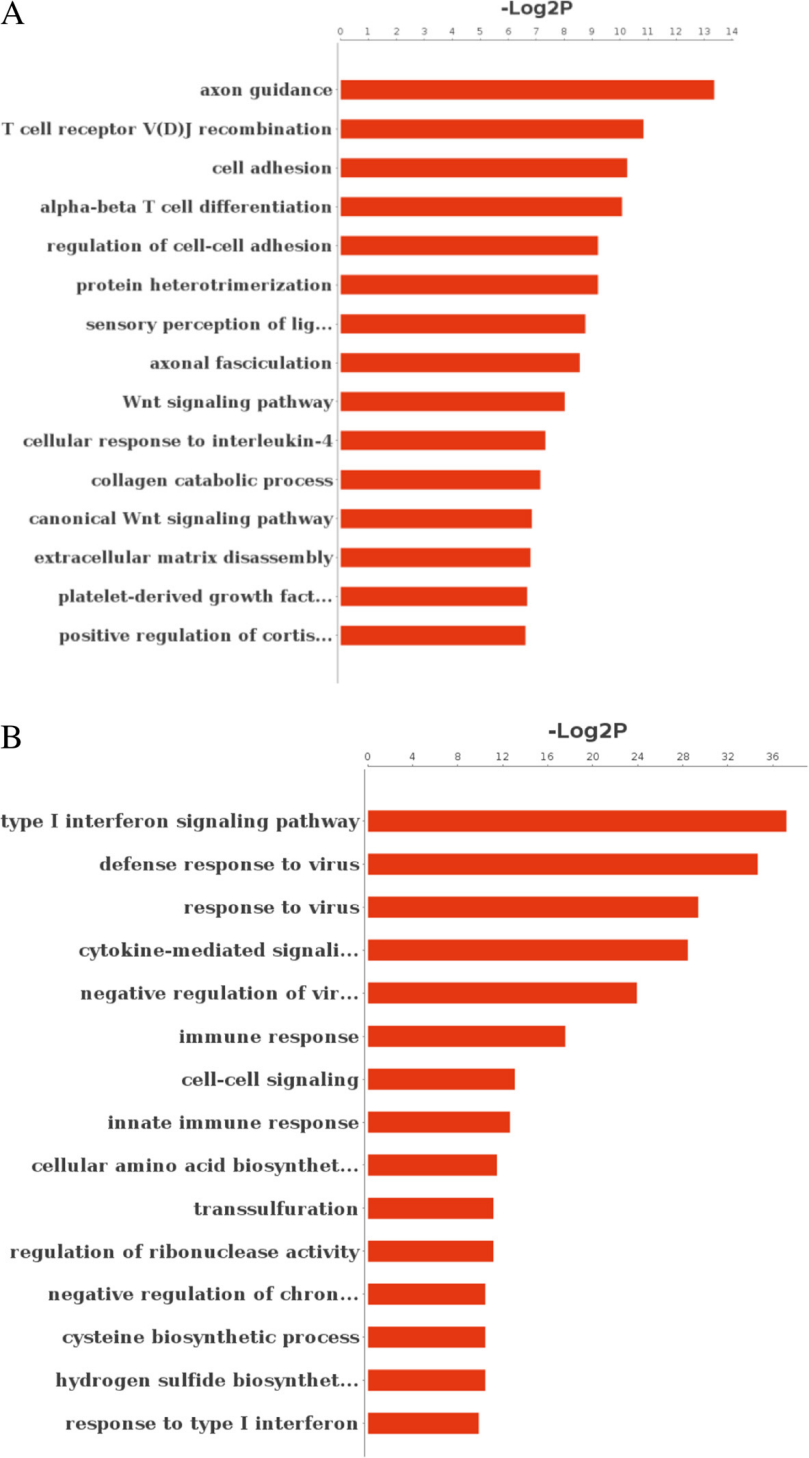
**Significant gene ontology analysis of DEGs in CIK**_**IL-15 **_**and CIK**_**IL-2**_**. (A)** Significant GO terms of CIK_IL-15_; **(B)** Significant GO terms of CIK_IL-2_. P value < 0.01 for all significant GO terms.

To characterize the functional consequences of gene expression changes induced by IL-15 and IL-2, we screened the differentially expressed genes (DEGs) between CIK_IL-15_ cells and CIK_IL-2_ cells by the following criteria: Log_2_FC > 1 or Log_2_FC < −1, FDR < 0.05 and P value < 0.05. We found 374 DEGs between CIK_IL-15_ and CIK_IL-2_ Additional file 2. Of these DEGs, 175 and 199 genes were up-regulated in CIK cells activated by IL-15 and IL-2 respectively. We used hierarchical cluster analysis to compare the DEGs between these two types of CIK cells and similarity of expression patterns of three biological replicates (Figure 2). To identify the functions of these DEGs, we performed gene ontological analysis based on GO database Additional file 3. Among these DEGs which were up-regulated in CIK_IL-15_, there were 11 genes involved in cell adhesion and 5 genes involved in Wnt signaling pathway (Figure 2). By analyzing the significant GO terms, we found that T cell receptor V(D)J recombination, cell adhesion and alpha-beta T cell differentiation were involved (Figure 3A). In order to target the DEGs which may cause functional changes, we screened DEGs whose GO terms were closely related with the functions of CIK cells. Based on the functional assay, CIK_IL-15_ cells have shown greater proliferation capacity than CIK_IL-2_ cells in vitro. Interestingly, we found that *Wnt 4* was significantly up-regulated in CIK_IL-15_ compared to CIK_IL-2_ (Table 2). By gene ontological analysis, *Wnt 4* is involved in multiple biological processes including Wnt signaling pathway, immature T cell proliferation and negative regulation of apoptosis (Table 2). Platelet-derived growth factor D (PDGFD) is a growth factor that plays an essential role in cell proliferation and survival. The expression of PDGFD is up-regulated after stimulation of IL-15. Therefore, we speculated that the enhanced proliferation capacity of CIK_IL-15_ may be brought by up-regulation of Wnt4 and PDGFD. Interleukin 21 receptor, which has played important role in natural killer cell activation and cytokine signaling pathway was found highly expressed in CIK_IL-15._ Moreover, E3 ubiquitin protein ligase (DTX4) and intercellular adhesion molecule (ICAM4) were also up-regulated in CIK_IL-15_. These proteins may be involved in type I interferon production and cell adhesion. Among the DEGs in CIK_IL-2_, there were 17 genes participated in innate immune response, 16 genes involved in cytokine-mediated signaling pathway and 12 genes involved in type I interferon signaling pathway (Figure 2). By analyzing the significant go terms, we found that type I interferon signaling pathway, cytokine-mediated signaling pathway and immune response are significant GO terms in response to stimulation of IL-2 (Figure 3B). Compared to CIK_IL-15_, CIK_IL-2_ has shown enhanced cytotoxic capacity against tumor. Consistently, we have found 3 tumor suppressive genes which were significantly up-regulated in CIK_IL-2_ including tumor necrosis factor ligand superfamily member 10 (TNFSF10), CD40 ligand (CD40LG) and interferon regulatory factor 7 (IRF7) (Table 3). These genes were widely involved in positive regulation of apoptotic signaling pathway, potent anti-tumor effect and promote type I interferon production. Surprisingly, we found that CD80 and its inhibitory ligand CTLA4 were co-upregulated in CIK cells after activation of IL-2. The function of CD80 is mainly involved in the costimulatory signal for T lymphocyte activation. CTLA4 functions as a negative regulator of T cell activation, which may inhibit the T cell proliferation.

**Table 2:** **Up-regulated genes related with functions and phenotypes of CIK**_**IL-15**_

**Gene symbol**	**Description**	**Log**_**2**_**FC**	**P value**	**FDR**	**Go Term**
**Wnt 4**	Protein Wnt-4	1.10	3.61 × 10^−4^	7.28 × 10^−3^	Regulation of cell-cell adhesion; Wnt signaling pathway; immature T cell proliferation in thymus; positive regulation of focal adhesion assembly; T cell differentiation in thymus; cell differentiation; cell-cell signaling; negative regulation of apoptotic process; positive regulation of transcription, DNA-templated
**IL21R**	Interleukin 21 receptor	1.17	2.53 × 10^−4^	5.53 × 10^−3^	Interleukin-21-mediated signaling pathway; natural killer cell activation; cytokine-mediated signaling pathway
**DTX4**	E3 ubiquitin-protein ligase	2.65	2.02 × 10^−3^	2.82 × 10^−2^	Regulation of type I interferon production; positive regulation of type I interferon production; Notch signaling pathway; innate immune response; protein ubiquitination
**ICAM4**	Intercellular adhesion molecule 4	1.90	1.46 × 10^−4^	3.51 × 10^−3^	Cell adhesion; cell-cell adhesion; regulation of immune response
**PDGFD**	Platelet-derived growth factor D	1.65	2.38 × 10^−5^	8.11 × 10^−4^	Platelet-derived growth factor receptor signaling pathway; cellular response to amino acid stimulus; multicellular organismal development; regulation of peptidyl-tyrosine phosphorylation; positive regulation of cell division

**Table 3:** **Up-regulated genes related with functions and phenotypes of CIK**_**IL-2**_

**Gene symbol**	**Description**	**Log**_**2**_**FC**	**P value**	**FDR**	**Go terms**
**CTLA4**	Cytotoxic T-lymphocyte-associated protein 4	1.01	1.64 × 10^−3^	2.38 × 10^−2^	Immune response; negative regulation of regulatory T cell differentiation; negative regulation of B cell proliferation; T cell costimulation; B cell receptor signaling pathway; cellular response to DNA damage stimulus; positive regulation of apoptotic process
**CD80**	CD80 antigen	1.03	1.11 × 10^−3^	1.75 × 10^−2^	Innate immune response; positive regulation of GMCSF biosynthetic process; positive regulation of T-helper 1 cell differentiation; T cell activation; regulation of interleukin-2 biosynthetic process; T cell costimulation
**TNFSF10**	Tumor necrosis factor ligand superfamily member 10	1.24	7.02 × 10^−18^	4.45 × 10^−15^	Immune response; activation of cysteine-type endopeptidase activity involved in apoptotic process regulation of extrinsic apoptotic; signaling pathway in absence of ligand; apoptotic process; positive regulation of extrinsic apoptotic signaling pathway; positive regulation of release of cytochrome c from mitochondria; apoptotic signaling pathway; positive regulation of cysteine-type endopeptidase activity involved in apoptotic process; positive regulation of apoptotic process
**CD40L**	CD40 ligand	2.08	2.32 × 10^−6^	1.18 × 10^−4^	Immune response; inflammatory response; immunoglobulin secretion; positive regulation of endothelial cell apoptotic process; B cell proliferation; positive regulation of interleukin-12 production; leukocyte cell-cell adhesion
**IRF7**	Interferon regulatory factor 7	1.08	3.12 × 10^−5^	1.02 × 10^−3^	Innate immune response; inflammatory response; positive regulation of type I interferon-mediated signaling pathway; positive regulation of type I interferon production; toll-like receptor signaling pathway

### Pathways analysis of CIK_IL-15_ and CIK_IL-2_

**Figure 4:**
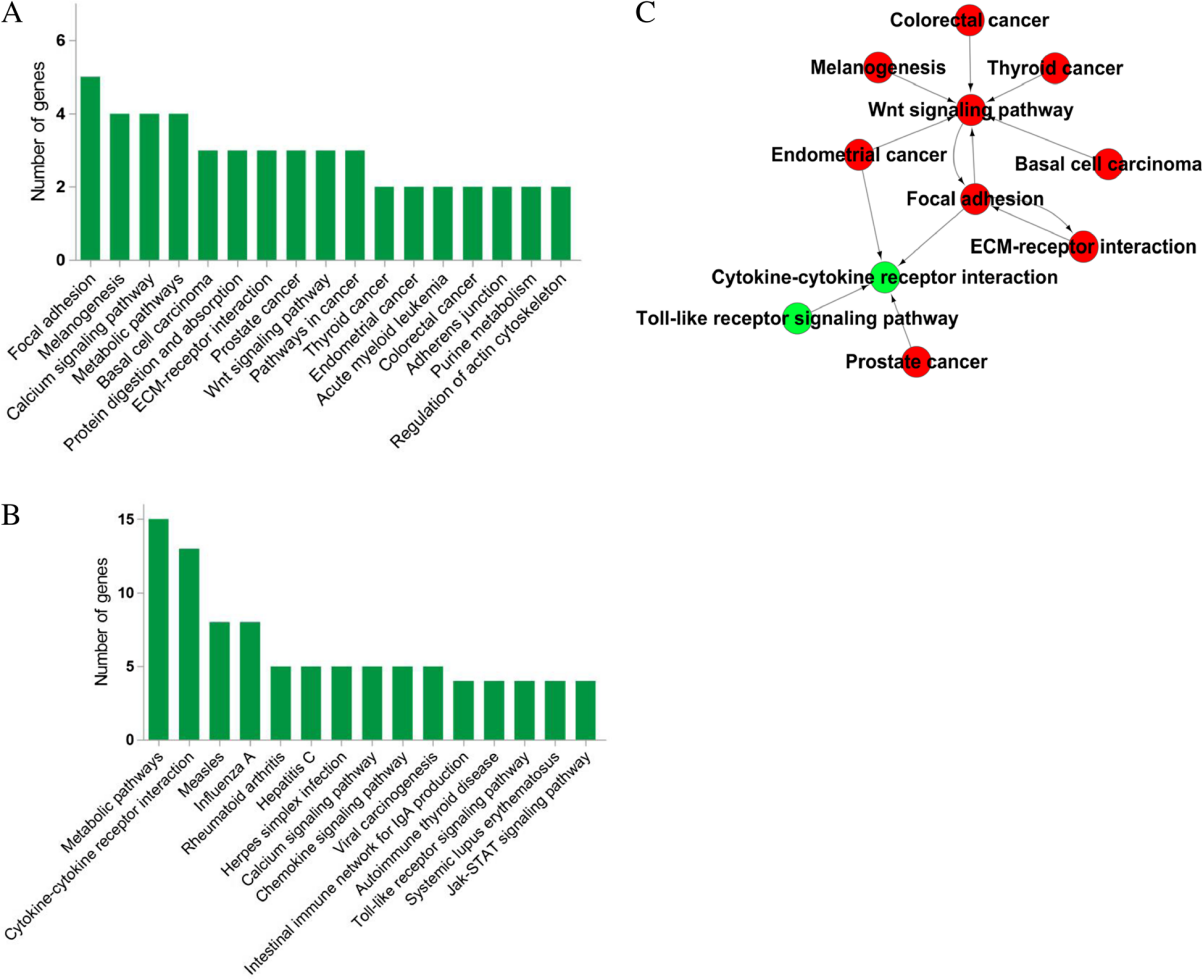
**Pathway enrichment analysis of DEGs based on KEGG. (A)** Enriched pathways in CIK_IL-15_; **(B)** Enriched pathways in CIK_IL-2_; **(C)** Pathways interaction network of CIK_IL-15_ and CIK_IL-2_, Red circles represent enriched pathways in CIK_IL-15_; Green circle represent enriched pathways in CIK_IL-2_.

To further identify the influence of DEGs on the functions of these two types of CIK cells, we performed pathway analysis of DEGs based on KEGG database using Fisher exact test Additional file 4. Among DEGs of CIK_IL-15_, there 5 genes participated in focal adhesion including collagen type VI alpha 3 (COL6A3), collagen alpha-2(VI) chain (COL6A2), collagen alpha-1(VI) chain (COL6A1), Platelet-derived growth factor D (PDGFD) and Myosin light chain kinase family member 4 (MYLK4) (Figure 4A). Surprisingly, 3 genes coding collagens were involved in this pathway which may be related with enhanced cell proliferation capacity of CIK_IL-15_. In CIK_IL-2_, the results indicated that 13 genes participated in cytokine-cytokine receptor interaction (Figure 4B). Of these genes, IL-4 and CXCL10 were newly identified DEGs that may contributed to tumor suppression. Subsequently, we have built the pathways interaction network to perform deep analysis. Through analyzing the interactions among the significant pathways, it was obvious Wnt signaling pathway, focal adhesion and cytokine-cytokine receptor interaction were the most important pathways involved in the function of CIK_IL-15_ and CIK_IL-2_ (Figure 4C). Because these three pathways located at the centers of each clusters and showed the most interactions with their surrounding pathways (the most arrows toward them). The results suggested that Wnt 4 signaling pathway and focal adhesion be the key biological events of CIK_IL-15_ cell proliferation, and cytokine-cytokine receptor interaction be the dominant element in CIK_IL-2_ cells in acquisition of tumor cytotoxic capacity. This evidence indicated that DEGs involved in these three pathways may play important roles in the distinct functions of CIK_IL-15_ and CIK_IL-2_.

### Differentially expressed genes act network

**Figure 5:**
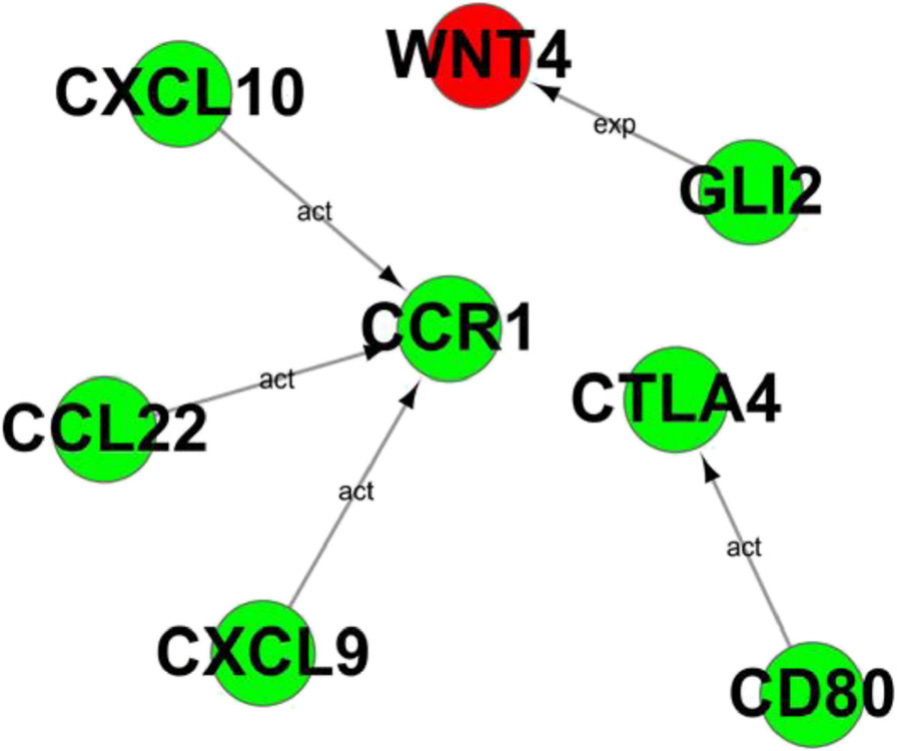
**Gene Act network analysis.** Red circles represent up-regulated genes in CIK_IL-15_; Green circles represent up-regulated genes in CIK_IL-2_.

After functional analysis, it is important to explore the relationships among these DEGs. According to KEGG database, we built the act network of genes based on the relationships between them including activation\binding, expression, inhibition and compound. In this gene interaction network, CXCL10, CXCL9, CCL22, GLI2, WNT4, CD80 and CTLA4 were in involved in pathways which previously mentioned including Wnt signaling pathway, Cytokine-cytokine receptor interaction and T cell signaling (Figure 5). In Figure 5, we showed that GLI2 (Zinc finger protein) functioned as transcription factor which involved in the expression of protein Wnt 4 in CIK_IL-15_. Again, the interaction between CD80 and CTLA-4 has been highlighted in CIK_IL-2_. After stimulation of IL-2, CD80 were up-regulated and interacted with CD28 providing costimulation signal for T cell activation and proliferation. However, negative feedback has been turned on through up-regulating the expression of CTLA4 which bound to CD80 providing inhibitory signal instead of CD28. Moreover, we also have noticed that CXCL10, CCL22 and CXCL9 were associated with CCR1. The association among these genes may be related with anti-tumor activity and CIK cell recruitment.

### Gene co-expression network

**Figure 6:**
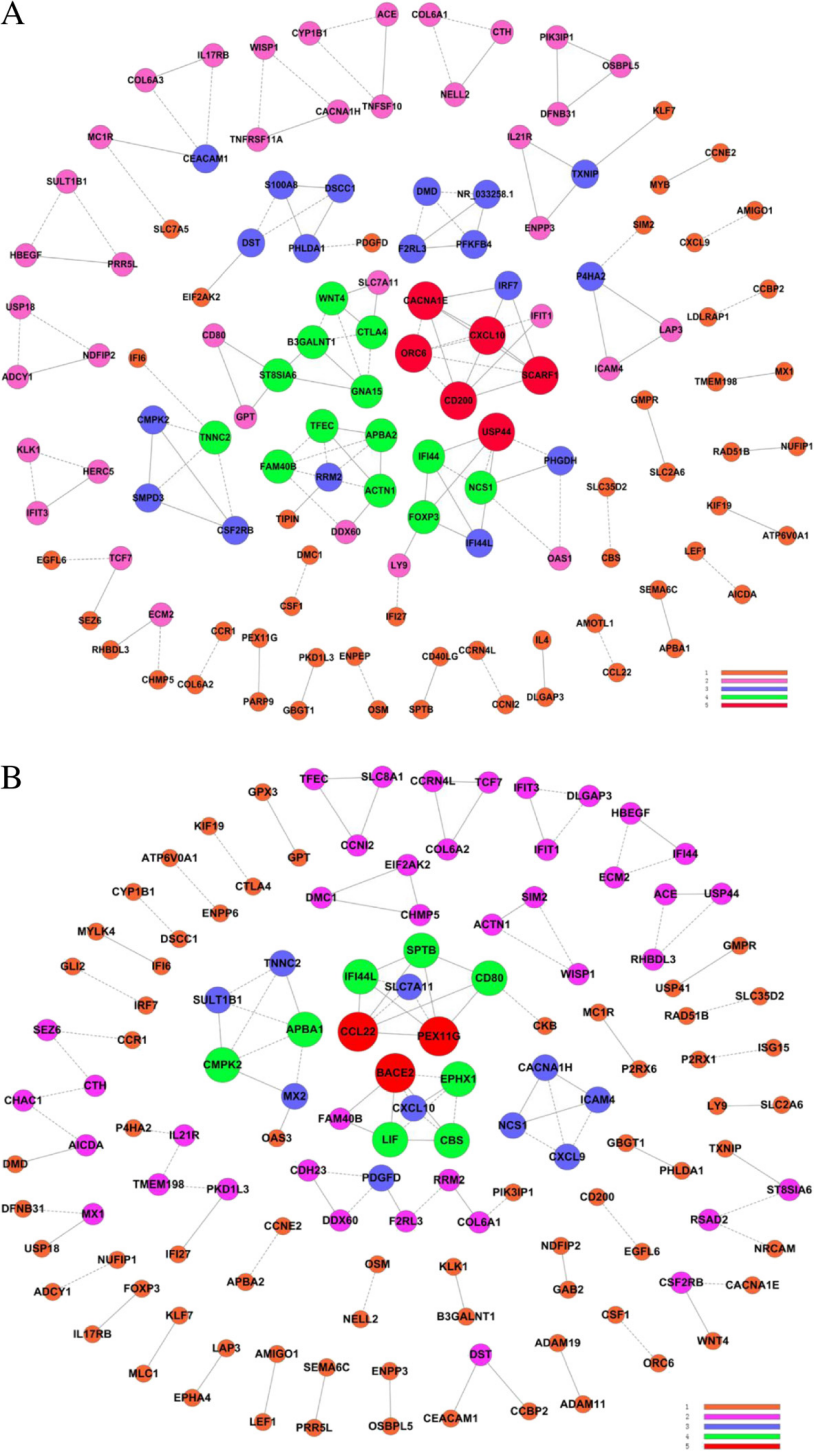
**Gene co-expression network analysis. (A)** CIK_IL-15_; **(B)** CIK_IL-2_; Degree in different color is defined as the link numbers one node has with the other. The Pearson Correlation of each pair of genes were calculated from these three independent samples.

Alternatively, we performed the gene co-expression network analysis between DEGs in CIK_IL-15_ and CIK_IL-2_ to highlight groups of DEGs in synergy which may participate in biological processes resulted in phenotypic changes [31,32]. Among DEGs of CIK_IL-15_, we showed that the expression levels of IL21R (Interleukin 21 receptor), ENPP3 (Ectonucleotide pyrophosphatase/phosphodiesterase 3) and TXNIP (Thioredoxin interacting protein) were positively correlated (Pearson’s r = 0.99), and mainly involved in immune response (Figure 6A). Moreover, we also found a group of genes that related with immune response including IFI44 (Interferon-induced protein 44), FOXP3 (Forkhead box P3), IF44L (Interferon-induced protein 44-like), LY9 (T-lymphocyte surface antigen Ly-9) and IFI27 (Interferon, alpha-inducible protein 27) in CIK_IL-15_ (Figure 6A). It was obvious that a cluster genes related with cell proliferation and apoptosis including PDGFD (Platelet-derived growth factor D), PHLDA1 (PHLDA1 protein), DSCC1 (Sister chromatid cohesion protein), S100A8 (Protein S100-A8), DST (Bullous pemphigoid antigen 1) and EIF2AK2 (EIF2AK2 protein) were correlated in CIK_IL-15_ (Figure 6A). In CIK_IL-2_, three pairs of genes with similar expression profiles were found to be involved in type I interferon signaling pathway (MX1/USP18; MX2/OAS1; IFT1/IFT3) (Figure 6B). Interestingly, the expression pattern of T cell activation negative regulator Foxp3 was correlation with the expression of IL-17 receptor B in CIK_IL-2_ (Figure 6B).

### Validation of representative genes by qRT-PCR

**Figure 7:**
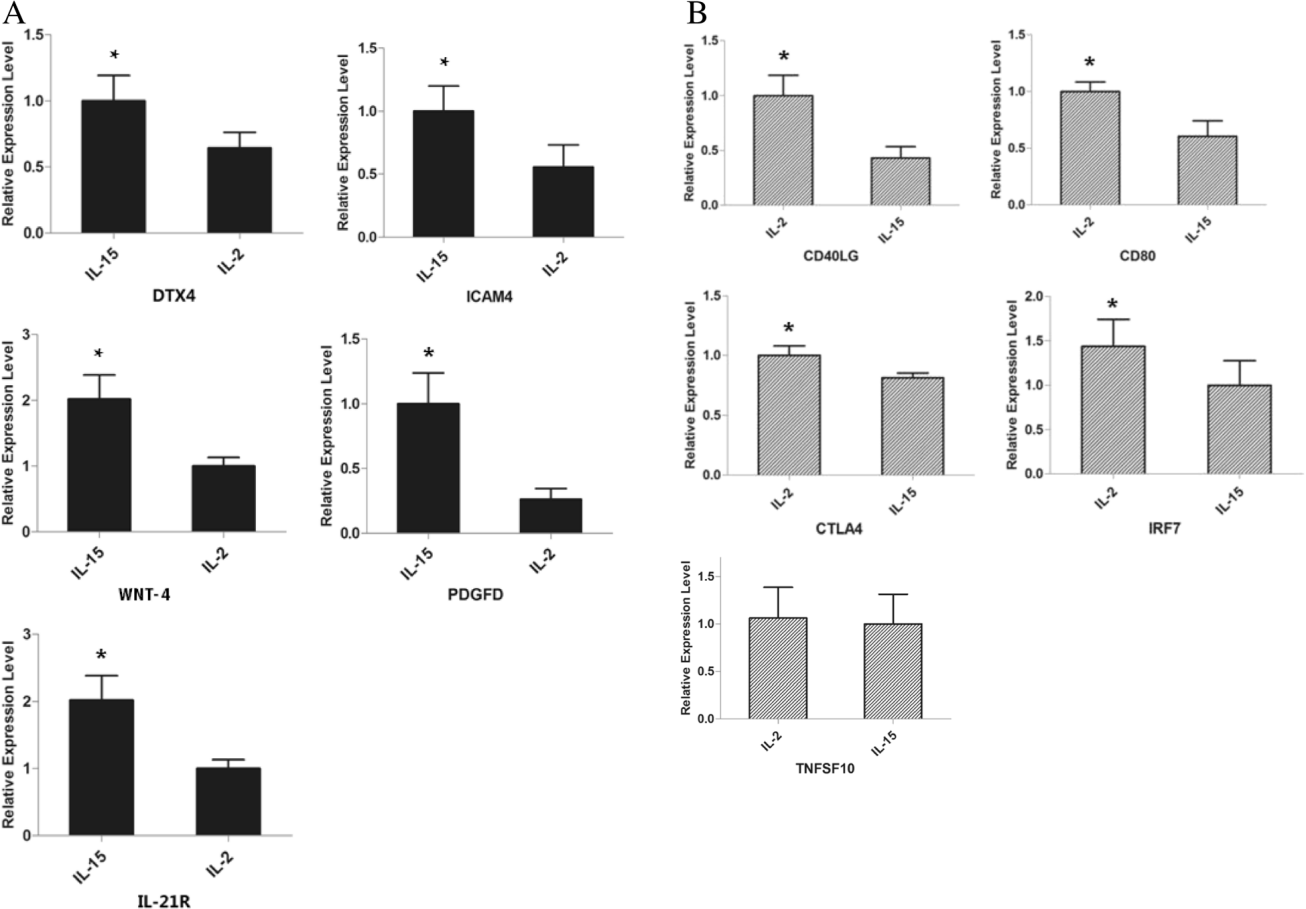
**qRT-PCR validation of relative expression levels of representative DEGs. (A)** DEGs in CIK_IL-15_; **(B)** DEGs in CIK_IL-2_; the expression levels of corresponding genes were normalized by β-actin. The results were means and SEMs, representative of nine independent samples. (P < 0.05 in all DEGs except TNSF10).

We have examined the expression profiles of DEGs which were referred in Table 2 and Table 3. The results of qRT-PCR have indicated that the expression profiles of DEGs in CIK_IL-15_ and CIK_IL-2_ were consistent with RNA-seq except for TNFSF10 (Figure 7). Notably, the expression level of Wnt 4 in CIK_IL-15_ was over 3 fold of those in CIK_IL-2_. However, TNFSF10 in CIK_IL-2_ were slightly higher than CIK_IL-15_ (p>0.05). Therefore, TNFSF10 may not be a contributor of enhanced tumor toxic function of CIK_IL-2_.

## Discussion

Although clinical trials of CIK cells in cancer therapy were widely performed in China, fewer studies on molecular mechanism of their anti-tumor function were observed [33,34]. The pioneering work of CIK cells was performed by Schmidt-Wolf from Stanford. The authors indicated that CIK cells were a subset of non-MHC-restricted T cells expressing both CD3 and CD56, and CIK cells showed potent cytotoxicity against a variety of tumor cells [2]. The efficiency of CIK cells preparation is dependent on T cell proliferation and cytolytic activity against tumor. To generate CIK cells with high quality, cytokines such as IL-1, IL-7, IL-15 and IL-12 have been employed instead of IL-2 or in combination with IL-2 [4]. Of these cytokines, IL-15 is widely tested in CIK cells preparation against several tumor cells. In this study, the results have indicated that CIK_IL-15_ exhibit enhanced proliferation capacity than CIK_IL-2_, whereas, CIK_IL-2_ showed more efficient cytotoxic effect against tumor cells than CIK_IL-15_. Consistently, the results from transcriptome analysis have shown corelationship with their functional characteristics.

IL-15 is a pleiotropic cytokine which promote T cells and NK cells proliferation and survival [35,36]. To better elucidate the mechanism of increased proliferation capacity induced by IL-15, we have found that Wnt 4 and PDGFD which were correlated with cell proliferation were up-regulated in CIK_IL-15_. Wnt signaling pathway is widely involved in cell proliferation and differentiation [37]. It has been reported that Wnt agonist promoted mouse muscle cell proliferation, and specific silencing RNA knockdown of Wnt 4 significantly reduced muscle cell proliferation [38]. Moreover, study showed that the expression of Wnt 4 was required for proliferation of cells in mouse coelomic epithelium [39]. By pathway interaction analysis, we have found that Wnt signaling pathway is located at the center of the network, which got the most interactions with other pathways. Therefore, we suggested that Wnt signaling be one of the most important pathways which contributed to the improved proliferation capacity of CIK_IL-15_. Except for Wnt 4, PDGFD is also a proliferation promoting factor which regulates several cellular processes including cell proliferation, apoptosis and transformation [40]. Over-expression of PDGFD in mouse or human breast cancer cell significantly increased cell proliferation while silencing PDGFD expression decreased proliferation and increased apoptosis [41]. Studies have indicated that PDGFD promoted cell proliferation by increasing DNA binding capacity of NF-κB and down-regulation of PDGFD inhibit tumor invasion through inactivation of Notch-1 and NF-κB signaling [42]. Therefore, the up-regulation of Wnt 4 and PDGFD may be responsible for enhanced cell proliferation of CIK_IL-15_.

Additionally, we also have found important evidence which may inhibit the proliferation of CIK_IL-2_. By differentiated expressed genes and gene act network analysis, we found that CTLA4 and CD80 were up-regulated in CIK_IL-2_. These two proteins can interact with each other to provide inhibitory signal during T cell activation [43]. In the generation of CIK_IL-2_, OKT3 and IL-2 were sustainedly presented in the culture system. However, IL-2 mediated activation-induced cell death (AICD) occurred during the following culture [7]. Consistently, our previous phenotypic study of CIK_IL-2_ have showed that the cells subset of CD3^+^CD28^+^ was increasing in the first several days while significantly decreased since the 7^th^ day of culture (Data not shown). These results demonstrated that the interaction between CTLA4 and CD80 may lead to inactivation of CD3^+^CD28^+^ T cell and inhibit proliferation of CIK_IL-2_. The inhibitory signal from ligation of CTLA4 to CD80 is the negative feedback to IL-2 stimulation of CIK cells. Comprehensively, not only up-regulated Wnt 4 and PDGFD but also activation inhibitory signal from CTLA4 and CD80 in CIK_IL-2_ has resulted in the enhanced proliferation capacity of CIK_IL-15_. We suggest that supplement with cytokines or mAb which down-regulates the inhibitory signal from CTLA4 and CD80 facilitate proliferation of CIK_IL-2_ production.

The most important of characteristic of CIK cell is cytolytic activity against tumor. In vitro, CIK_IL-2_ cells have shown more efficient tumor cytotoxicity than CIK_IL-15_. The expression of CD40LG and IFR7 were up-regulated in CIK_IL-2_. CD40LG, which is the ligand of CD40, has shown great potentials in cancer therapy [44]. It has been reported that CD40 is expressed in nearly all B cell malignancy and many solid tumors [45]. The ligation of CD40 on the surface of tumor cells inhibits the growth of tumor and induces apoptosis [46]. Besides CD40LG, IFN-β has also been found to play critical role in anti-tumoral immune response [47]. Interestingly, Moschonas has indicated that stimulation of CD40 by its ligand has promoted the expression of IFN-β through the binding IRF7 to its promoter. IRF7 is a transcriptional factor which regulates the expression of type I interferon [48]. Silencing of IRF7 pathways in breast cancer accelerated bone metastasis through immune escape [49]. Thus CD40LG and IFR7 may work synergically to improve the tumor cytotoxic effect of CIK_IL-2_.

On the other hand, the expression of DTX4 was up-regulated in CIK_IL-15_ which positively regulated the production of type I interferon through NLRP4 [50]. Moreover, the expression of IL-21R whose ligand is involved in natural killer cell was also increased in CIK_IL-15_. Paradoxically, the up-regulation of PDGFD in CIK_IL-15_ not only could promote the proliferation of CIK_IL-15_ cells but also promote tumor cells survival through cell and cell interaction in tumor cytotoxic assay. PDGFs are composed of four different polypeptide chains (PDGF A-D). It has been reported that PDGFD was deregulated in most of human malignancies with up-regulated expression in solid tumors [51]. The factor interacts with PDGFR-β and activates downstream signaling phosphatidylinositol 3-kinase (PI3K)/AKT, resulted in tumor progression. Moreover, Li et al. reported that PDGFD is a potent transformation growth factor for NIH/3 T3 which increased the cell proliferation rate [52]. We suggested that up-regulated PDGFD is a double-edged sword in CIK_IL-15_. Because it favored the proliferation of CIK_IL-15_ cells during preparation, while it may also promoted the survival and proliferation of tumor cells when CIK cells were in contact with tumor cells.

## Conclusions

In this study, deep sequencing was performed to analyze the different gene expression profiles of CIK_IL-2_ and CIK_IL-15_ for the first time. By advanced bioinformatic analysis of DEGs, we found that cell proliferation promoting function was dominant in CIK_IL-15_ involving Wnt signaling pathway and focal cell adhesion. In CIK_IL-2_, type I interferon signaling pathway and cytokine-cytokine receptor interactions were dominant. Through our findings, we have preliminarily elucidated the cells proliferation and acquisition of tumor cytotoxicity mechanism of CIK_IL-15_ and CIK_IL-2_. Better understanding of these mechanisms will help to generate novel CIK cells with greater proliferation potential and improved tumor cytolytic function.

## Competing interests

The authors declare that they have no competing interests.

## Authors’ contributions

ZLH and RHL conceived and designed the study. WJW, MYM and YYZ performed the experiments. WJW, DC and JZ analyzed the data. CHW and WWT contributed reagents/material. CYW, YHX and LHJ interpreted the data and wrote the paper. FY and XFJ revised the manuscript. All authors read and approved the final manuscript.

## Pre-publication history

The pre-publication history for this paper can be accessed here:

http://www.biomedcentral.com/1755-8794/7/49/prepub

## Supplementary Material

Additional file 1: Table S1 RNA-seq of data of all count for CIK_IL-15_ and CIK_IL-2_. Click here for file

Additional file 2: Table S2 The list of differentially expressed genes between CIK_IL-15_ and CIK_IL-2_. Click here for file

Additional file 3: Table S3 GO analysis differentially expressed genes in CIK_IL-15_ and CIK_IL-2_. Click here for file

Additional file 4: Table S4 Pathway analysis differentially expressed genes in CIK_IL-15_ and CIK_IL-2_. Click here for file
